# Sphingomyelin(d35:1) as a novel predictor for lung adenocarcinoma recurrence after a radical surgery: a case-control study

**DOI:** 10.1186/s12885-020-07306-1

**Published:** 2020-08-24

**Authors:** Yusuke Takanashi, Kazuhito Funai, Shumpei Sato, Akikazu Kawase, Hong Tao, Yutaka Takahashi, Haruhiko Sugimura, Mitsutoshi Setou, Tomoaki Kahyo, Norihiko Shiiya

**Affiliations:** 1 Department of Cellular and Molecular Anatomy, Hamamatsu University School of Medicine, 1-20-1 Handayama, Higashi Ward, Hamamatsu, Shizuoka, 431-3192 Japan; 2 First Department of Surgery, Hamamatsu University School of Medicine, 1-20-1 Handayama, Higashi Ward, Hamamatsu, Shizuoka, 431-3192 Japan; 3 Department of Tumor Pathology, Hamamatsu University School of Medicine, 1-20-1 Handayama, Higashi Ward, Hamamatsu, Shizuoka, 431-3192 Japan; 4 Preppers Co. Ltd., 1-23-17 Kitashinagawa, Shinagawa Ward, Tokyo, 140-0001 Japan; 5 International Mass Imaging Center, Hamamatsu University School of Medicine, 1-20-1 Handayama, Higashi Ward, Hamamatsu, Shizuoka, 431-3192 Japan; 6 Department of Systems Molecular Anatomy, Institute for Medical Photonics Research, Hamamatsu University School of Medicine, 1-20-1 Handayama, Higashi Ward, Hamamatsu, Shizuoka, 431-3192 Japan

**Keywords:** Lung adenocarcinoma, Prognostic factor, Recurrence prediction, Lipid, Mass spectrometry

## Abstract

**Background:**

To improve the postoperative prognosis of patients with lung cancer, predicting the recurrence high-risk patients is needed for the efficient application of adjuvant chemotherapy. However, predicting lung cancer recurrence after a radical surgery is difficult even with conventional histopathological prognostic factors, thereby a novel predictor should be identified. As lipid metabolism alterations are known to contribute to cancer progression, we hypothesized that lung adenocarcinomas with high recurrence risk contain candidate lipid predictors. This study aimed to identify candidate lipid predictors for the recurrence of lung adenocarcinoma after a radical surgery.

**Methods:**

Frozen tissue samples of primary lung adenocarcinoma obtained from patients who underwent a radical surgery were retrospectively reviewed. Recurrent and non-recurrent cases were assigned to recurrent (*n* = 10) and non-recurrent (n = 10) groups, respectively. Extracted lipids from frozen tissue samples were subjected to liquid chromatography-tandem mass spectrometry analysis. The average total lipid levels of the non-recurrent and recurrent groups were compared. Candidate predictors were screened by comparing the folding change and *P*-value of t-test in each lipid species between the recurrent and non-recurrent groups.

**Results:**

The average total lipid level of the recurrent group was 1.65 times higher than that of the non-recurrent group (*P* < 0.05). A total of 203 lipid species were increased (folding change, ≥2; P < 0.05) and 4 lipid species were decreased (folding change, ≤0.5; P < 0.05) in the recurrent group. Among these candidates, increased sphingomyelin (SM)(d35:1) in the recurrent group was the most prominent candidate predictor, showing high performance of recurrence prediction (AUC, 9.1; sensitivity, 1.0; specificity, 0.8; accuracy, 0.9).

**Conclusion:**

We propose SM(d35:1) as a novel candidate predictor for lung adenocarcinoma recurrence. Our finding can contribute to precise recurrence prediction and qualified postoperative therapeutic strategy for lung adenocarcinomas.

**Trial registration:**

This retrospective study was registered at the UMIN Clinical Trial Registry (UMIN000039202) on 21st January 2020.

## Background

Lung cancer is one of the leading causes of cancer-related mortality worldwide. Radical resection is the standard treatment for stage I–II non-small cell lung cancer (NSCLC) [1]. However, the postoperative survival rate remains unsatisfactory despite complete resection. Among patients with NSCLC who received complete resection, 23.9% experience local or distant disease recurrence [2]. Therefore, adjuvant chemotherapy should be administered to improve survival after a radical surgery [3].

Adjuvant chemotherapy has been shown to reduce the risk of death due to lung cancer recurrence [4–7]. Nonetheless, not all patients who underwent radical surgery benefit from adjuvant chemotherapy, because some of them are already successfully healed without adjuvant chemotherapy. Therefore, patients highly at risk for recurrence who are likely to benefit from adjuvant chemotherapy should be identified for the efficient application of adjuvant chemotherapy.

Adenocarcinoma is the most common histological type of NSCLC, accounting approximately 80% of all NSCLC cases [8]. In lung adenocarcinomas, several prognostic factors obtained by histopathological evaluations of surgical specimens have been reported to date, such as lymph node metastasis [9], pleural invasion [10], lymphatic vessel invasion [8, 11], blood vessel invasion [12, 13], adenocarcinoma subtype of micropapillary pattern [14], and spread through air space (STAS) [15, 16]. However, predicting lung cancer recurrence after radical surgery is still difficult, because data on the direct relationship between conventional prognostic factors and recurrence are limited. Furthermore, subjective judgments of conventional prognostic factors are considered to hinder accurate recurrence prediction and its retrospective validation. Accordingly, novel recurrence predictors with high objectivity are strongly expected.

Previous studies demonstrated that lipid metabolism alterations in cancer contribute to cancer cell proliferation and invasion [17, 18], and some lipids have been suggested as prognostic factors in several cancer types. For example, the number of phosphatidylcholine (PC)(32:1) in recurrent cases of primary triple-negative breast cancer (TNBC) is higher than in that of non-recurrent cases, and thereby, PC (32:1) is suggested as a candidate predictor for TNBC recurrence [19]. Oleic acid attenuation is correlated with shorter progression-free period in clear cell renal carcinoma [20]. With regard to lung cancer, although NSCLC is reportedly characterized by drastic changes in phospholipid profiles as compared to the normal lung tissue and contains different lipid profiles according to the histologic subtypes [21], no lipidomic approach to investigate the prognostic factor for NSCLC has been used. Based on these previous studies, we hypothesized that lung adenocarcinomas with high recurrence risk have different lipidomes from that of lung adenocarcinomas with low recurrence risk and specific lipids that can be considered as candidates as novel predictive factors for recurrence.

In this study, lipid species that can be considered as potential predictors for lung adenocarcinoma recurrence after a radical surgery were identified by comparing lipidomes of primary lung adenocarcinomas between recurrent and non-recurrent cases using liquid chromatography–tandem mass spectrometry (LC–MS/MS).

## Methods

### Patients and tissue samples

Retrospective frozen tissue samples of primary lung adenocarcinoma obtained from patients who underwent radical surgery from January 2013 to December 2016 at Hamamatsu University Hospital were examined. Radical surgery was defined as complete resection performed with lobectomy or pneumonectomy accompanied by systematic lymph node dissection at stage I or II, and as complete resection achieved by segmentectomy or wedge resection with or without lymph node sampling at stage I. Tissue samples of primary tumors were collected immediately after the resection and stored at − 80 °C after a rapid freezing in liquid nitrogen. Histopathological diagnosis was performed by experienced pathologists according to the World Health Organization criteria. Pathological staging was identified based on the 8th edition of the TNM classification for lung and pleural tumors [22]. Patients were followed-up with computed tomography (CT) of the body trunk and biochemical examination of carcinoembryonic antigen (CEA) every 3 months during the first 2 years, then, every 6 months until more than 5 years after the surgery. When CEA was elevated (≥5.0 ng/mL) without any CT findings of recurrence, head magnetic resonance imaging, and systemic positron emission tomography were performed for the detection of brain metastasis or bone metastasis.

In patient selection, clinical records of these tissue samples were retrospectively reviewed. Patients with pathological stage I or II indicated for radical surgery and with major histological subtypes of invasive adenocarcinoma (lepidic, papillary, acinar or solid predominant) were analyzed. Patients who received induction chemotherapy or radiotherapy and those with other subtypes of adenocarcinoma were excluded.

Then, cases without and with recurrence were assigned to non-recurrent and recurrent groups, respectively. Recurrence was defined as radiological imaging-based findings of distant or locoregional recurrence within 5 years, whereas no recurrence was defined as no findings of distant or locoregional recurrence in ≥5 years after the radical surgery. In the non-recurrent group, cases with follow-up period of < 5 years were excluded. In the recurrent group, cases with recurrence in the form of pleural dissemination were excluded, assuming the possible attribution with insufficient surgical margin. Finally, 10 cases for recurrent and 10 for non-recurrent groups were subjected for analysis.

### Histological evaluation

Paraffin-embedded tissue blocks were sectioned at 3 μm thick. Sections stained by hematoxylin–eosin (HE) were examined for adenocarcinoma subtype, tumor size, lymph node metastasis, and STAS. D2–40 stain was used to evaluate lymphatic vessel invasion and Elastica van Gienson stain to evaluate blood vessel invasion. All histological sections were reviewed by experienced pathologists.

### Chemicals

Methanol, chloroform, glacial acetate, and ultrapure water were purchased from Wako Pure Chemical Industries (Osaka, Japan). The 1,2-dilauroyl-sn-glycero-3-PC (Avanti Polar Lipids, Alabaster, AL), PC (12:0_12:0), was used to calibrate standard lipid levels.

### Lipid extraction from the cancer tissue

Each weight of the frozen tissue samples was measured using Sartorius analytical lab balance CPA224S (Sartorius AG, Göttingen, Germany) (Additional file 1, Supplemental Table). After the weight measurement, Modified Bligh-Dyer methods were performed for lipid extraction. Tissue samples were transferred into glass tubes, and 0.34 ml of methanol, 0.17 ml of chloroform, and 0.14 ml of 0.322 M glacial acetate were subsequently added. Then, 1.6 mmol of PC (12:0_12:0) per 1 mg of sample tissue was added and subsequently followed by 10-min extraction at room temperature. After the extraction, 0.17 ml of chloroform was added and vortexed, sequentially, 0.17 ml of 0.322 M glacial acetate was added and vortexed. Extracted samples were subjected to centrifugation at 3000 rpm for 10 min. Extracted organic layers were transferred into new glass tubes and were evaporated until completely dried using miVac Duo LV (Genevac, Ipswich, England). The extracted lipid was dissolved with 20 μl of methanol, and 2 μl of the dissolved lipids were diluted again with methanol proportional to the weight of the original tissue samples so that the concentration of PC (12:0_12:0), internal control, will be as similar as possible among cases.

### Lipid analysis by liquid chromatography–tandem mass spectrometry (LC–MS/MS)

Extracted lipids from collected frozen tissue samples were analyzed using Q Exactive™ Hybrid Quadrupole-Orbitrap™ Mass Spectrometer equipped with an electrospray ionization source and connected to an Ultimate 3000 system (Thermo Scientific). 10 μL of the extracted lipid samples were injected and separated on Acculaim 120 C18 column (150 mm × 2.1 mm, 3 μm) (Thermo Scientific). Components of mobile phase A were as follows: water-acetonitrile-methanol (2:1:1 v/v/v), 5 mM ammonium formate, and 0.1% formic acid. The components of mobile phase B were as follows: acetonitrile-isopropanol (1:9 v/v), 5 mM ammonium formate, and 0.1% formic acid. For elution, the flow rate was set at 300 μL/min. A set of linear gradient starting at 20% solvent B was used and linearly increased to 100% B in 50 min, maintained at 100% B until 60 min, then decreased linearly to 20% B from 60 min to 60.1 min, and finished with 20% B for the last 10 min. The overall run time was 70 min. MS instrument conditions were as follows: sheath gas flow rate, 50; auxiliary flow rate, 15; sweep gas flow rate, 0; capillary temperature, 250 °C; S-lens RF level, 50; probe heater temperature, 350 °C; and spray voltage of 3.5 kV in positive mode and 2.5 kV in negative mode. Full-MS mode conditions for quantification were as follows: MS scan range, 220–2000; resolution, 70,000; AGC target, 1 × 106 and maximum injection time was 100 ms. For identification, top 5 data-dependent MS2 method with a resolution of 17,500 was used. The AGC target was 1 × 105, and the maximum injection time was 80 ms. Stepped normalized collision energies of 25.5, 30, and 34.5 for the positive mode and 19.5, 30, and 40.5 for the negative mode were applied. Spectral data were acquired in the *m/z* range of 220–2000 *m/z* using an Xcalibur v3.0 Software (Thermo Scientific).

### Lipid identification and quantification

LipidSearch™ software version 4.2.13 (Mitsui Knowledge Industry, Tokyo, Japan) was used to identify and quantify lipid species. Parameter settings for identification were followings: database, HCD; retention time, 0.01 min; search type, product_QEX; precursor tolerance, 5.0 ppm; and product tolerance, 8.0 ppm. Identification quality filters of A, B, and C were used. Quantification was performed at *m/z* tolerance of ±0.01 with retention time range from − 1.0 min to 2.0 min. Alignment of the identified lipid species among 20 cases was performed with retention time tolerance of 0.25. Molecules that are annotated as redundant lipid names with different calculated *m/z* and retention times were regarded as independent isomers (annotated as “Duplication” in Additional file 2).

### Data processing

Trend analysis between the non-recurrent and recurrent groups was performed by comparing the average total lipid level between the two groups and principal component analysis (PCA). Intensities of lipids recorded in the Xcalibur v3.0 software and monoisotopic peak area values of lipid species identified by LipidSearch™ software were normalized by dividing with the area values of internal control, PC (12:0_12:0). The total lipid level of each case was defined as an accumulation of normalized intensities of lipids. Normalized area values were subjected for PCA.

For respective lipid species, *P*-values were calculated using the Student t-test to compare area values between the two groups. To screen candidate lipids for recurrence prediction, lipidomes were compared between the non-recurrent and recurrent groups by describing volcano plots with -log10 (*P*-value) for vertical axis and log2 (folding change) for horizontal axis. The folding change for a lipid was defined as an average area value of the recurrent group divided by that of the non-recurrent group. Significance was determined at *P*-values of < 0.05, folding change of ≥2.0 or ≤ 0.5.

### Statistical analysis

Demographic information and associations with clinical characteristics were evaluated using the Fisher exact test (categorical variables) or the Mann–Whitney U-test (for continuous variables). The Student t-test was used to compare the average total lipid amounts of the non-recurrent and recurrent groups and to describe volcano plots. Recurrent-free survival (RFS) was determined as the time from operation until the first disease recurrence or death. Survival curve was described using the Kaplan–Meier method. The optimal cut-off values to discriminate the two groups were determined using the receiver operating characteristic (ROC) curve analysis. The area under the ROC curves (AUCs) were calculated to validate the discrimination abilities of candidate lipids. Spearman’s rank correlation analysis was used to validate the correlation among candidate lipid predictors. All statistical analyses except for the t-test were performed using R (The R Foundation for Statistical Computing, Vienna, Austria, version 3.6.2). The Student t-test was performed with “TTEST” of Excel™ (Microsoft, Redmond, USA). *P*-values of < 0.05 were considered as significant.

## Results

### Clinicopathological characteristics of patient cohort

Clinicopathological characteristics of patients are shown in Table 1. In this study cohort, tissue samples from 10 non-recurrent and 10 recurrent cases were analyzed. Among the characteristics of these two groups, differences in pathological stage (*P* = 0.033), lymph node metastasis (P = 0.033), and blood vessel invasion (*P* = 0.005) were statistically significant. The 1- and 2-year RFS rate of the recurrent group was 50 and 20% with median RFS time of 12.5 (range, 9–38) months, respectively (Additional file 1, Supplemental Fig. 1). The median follow-up time of the non-recurrent and recurrent groups was 68.5 (range, 60–77) and 42.5 (range, 21–60) months, respectively.

**Table 1:** Clinicopathological characteristics of the non-recurrent and recurrent groups

Characteristics	Non-recurrent (n = 10)	Recurrent (*n* = 10)	*P*-value
Median age (range)	67.5 (49–75)	71.5 (67–89)	0.069
Gender (male/female)	7/3	7/3	1.000
Smoking history (+/−)	7/3	6/4	1.000
Pathological stage (I/II)	10/0	5/5	0.033
Median tumor size (mm) (range)	23.5 (9–29)	24 (9–37)	0.649
Adenocarcinoma subtype			0.293
Lepidic	2	0	
Papillary	5	7	
Acinar	1	2	
Solid	2	1	
Lymph node metastasis (+/−)	0/10	5/5	0.033
Pleural invasion (+/−)	2/8	5/5	0.350
Lymphatic vessel invasion (+/−)	1/9	6/4	0.057
Blood vessel invasion (+/−)	2/8	9/1	0.005
Micropapillary component (+/−)	5/5	8/2	0.350
Spread through air space (+/−)	2/8	4/6	0.628
Driver gene mutation			
EGFR (+/−)	2/8	6/4	0.170
ALK (+/−)	0/8	0/7	–
Surgical procedure			1.000
Lobectomy	10	9	
Wedge resection	0	1	
Adjuvant chemotherapy			1.000
Indication (Stage IA3-IIB)	8	9	
Received	4	4	
Recurrent style			–
Locoregional	–	3	
Distant	–	8	

Abbreviations: *ALK* anaplastic lymphoma kinase, *EGFR* epithelial growth factor receptor

### Trend analysis between the non-recurrent and recurrent groups

**Fig. 1:**
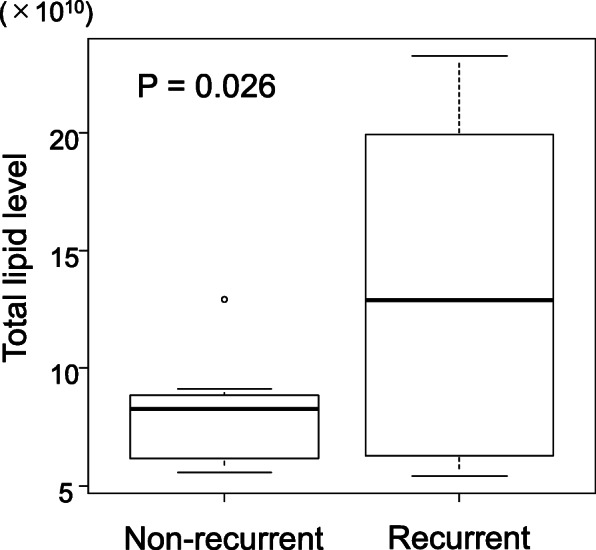
Comparison of total lipid levels between the recurrent and non-recurrent groups. The average total lipid level of the recurrent group was 1.65 times higher than that of the non-recurrent group (*P* = 0.026)

The frozen tissue samples were subjected to LC–MS/MS, and the total lipid level of cases was calculated by accumulating normalized intensities of lipids. Notably, the average total lipid level of the recurrent group was 1.65 times higher than that of the non-recurrent group (*P* = 0.026) (Fig. 1). A total of 2595 lipid species were identified and quantified by analyzing the mass spectral data using a LipidSearch™ software (the full list of identified 2595 lipid species is presented as Additional file 2), which were also subjected to PCA. The PCA plot did not show clear separation between the recurrent and non-recurrent groups; however, the recurrent group exhibited partial separations between the first three principal components (Additional file 1, Supplemental Fig. 2). These results suggested differences of lipidome between the recurrent and non-recurrent groups, which urged us to screen lipids to distinguish the two groups.

### Screening of candidate lipids for recurrence prediction

**Fig. 2:**
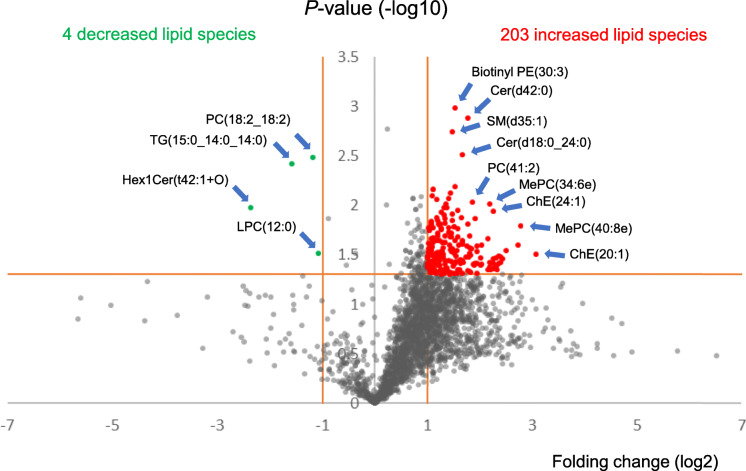
Volcano plots of 2595 identified lipid species. Each plot represents a lipid species to be identified. The relative amount of 203 lipid species (red plots) were increased (FC ≥ 2.0 = right side of 1 in the horizontal axis, *P*-value < 0.05 = 1.30 in vertical axis) and that of 4 lipid species (green plots) were decreased (FC ≤0.05 = left side of − 1 in the horizontal axis, *P*-value < 0.05 = 1.30 in vertical axis) in the recurrent group. Nine increased lipids showing prominent distributions and all 4 decreased lipid species were annotated for candidate predictors (blue arrows). Abbreviations: Cer, ceramide; ChE, cholesterol ester; FC, folding change; Hex1Cer, monohexosylceramide; LPC, lysophosphatidylcholine; MePC, monoether phosphatidylcholine; PC, phosphatidylcholine; PE, phosphoethanolamine; SM, sphingomyelin; TG, triglyceride

To screen lipids with different levels between the two groups, volcano plots of the identified lipids were described first, and lipidomes between the non-recurrent and recurrent groups were compared (Fig. 2). The volcano plot identified 207 lipid species, with relative amounts significantly different between the two groups (folding change, ≥2.0 or ≤ 0.5; *P*-values, < 0.05). The number of lipids that increased and decreased in the recurrent group was 203 and 4, respectively. These increased or decreased lipid species consisted of various head groups (Additional file 2, increased lipid species; shown in red, decreased lipid species; shown in green). Then, based on prominent distributions of the volcano plot, we narrowed the 203 candidate lipids increased in the recurrent group to the following 9 molecules (Fig. 2, blue arrows pointing to red plots): biotinyl-phosphoethanolamine (BiotinylPE)(30:3), ceramide (Cer)(d42:0), sphingomyelin (SM)(d35:1), Cer(d18:0_24:0), PC (41:2), monoether phosphatidylcholine (MePC)(34:6e), cholesterol ester (ChE)(24:1), MePC (40:8e), and ChE(20:1). As for the lipids that decreased in the recurrent group, the following four molecules were annotated (Fig. 2, blue arrows pointing to green plots): monohexosylceramide (Hex1Cer)(t42:1 + O), triglyceride (TG)(15:0_14:0_14:0), PC (18:2_18:2), and lysophosphatidylcholine (LPC)(12:0).

**Fig. 3:**
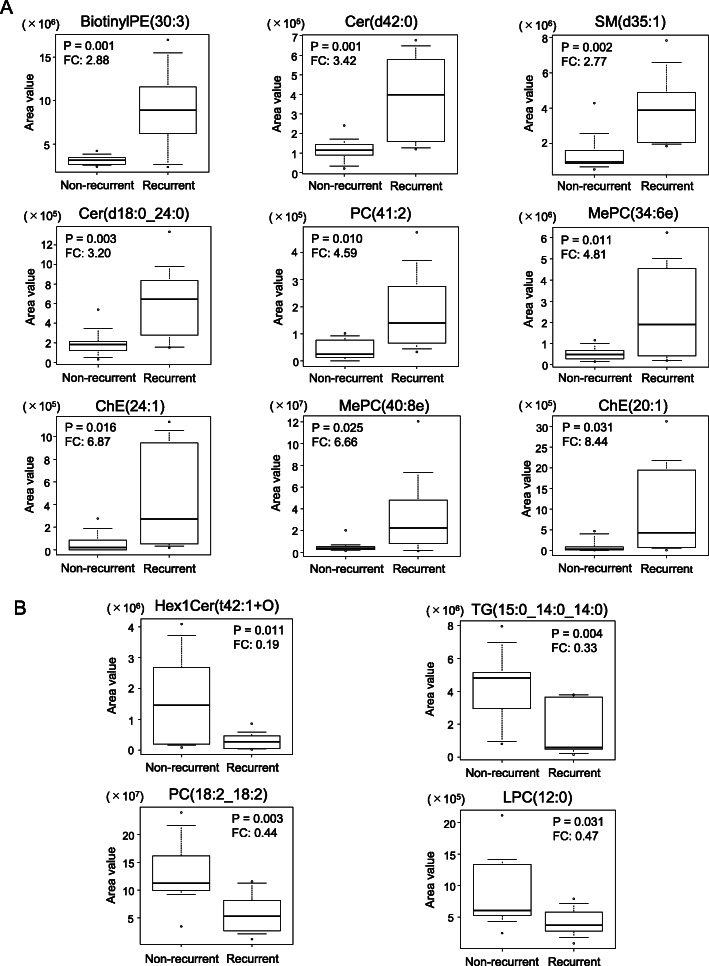
Comparisons of relative amount distributions between the non-recurrent and recurrent groups are shown for increased (**a**) and decreased (**b**) lipid species in the recurrent group. Boxplots show the upper 10 percentile, upper quartile, median, lower quartile, and lower 10 percentile. Maximum and minimum values are shown in dots. *P*-values for significance and FCs are presented for each lipid species. Abbreviations: Cer, ceramide; ChE, cholesterol ester; FC, folding change; Hex1Cer, monohexosylceramide; LPC, lysophosphatidylcholine; MePC, monoether phosphatidylcholine; PC, phosphatidylcholine; PE, phosphoethanolamine; TG, triglyceride

The relative amounts of these lipid species were evaluated with their distributions by comparing the two groups (Fig. 3a and b). In all tested lipids, distributions between the two groups were well separated enough to establish the cut-off values, whereas only few marked outliers were found.

We next calculated the cut-off values and AUC of these 13 lipids to evaluate their discrimination ability for disease recurrence, and the following final candidates with top three AUC were selected: SM(d35:1), 0.90; Cer(d42:0), 0.90; and TG (15:0_14:0_14:0), 0.90 (Table 2) (respective lipid species can be found in Additional file 2 with the following identical numbers: SM(d35:1), 2201; Cer(d42:0), 122; and TG (15:0_14:0_14:0), 2354). These three final lipid candidates were annotated as the following ions [SM(d35:1) + H]+, [Cer(d42:0) + HCOO]-, and [TG (15:0_14:0_14:0) + NH4] + in the LipidSearch™ software (Additional file 2).

**Table 2:** AUC rank of candidate lipid predictors determined by ROC curve

Rank*	Species	Cutoff value	AUC (95% CI)
**1**	**SM(d35:1)**	**1,866,710.893**	**0.91 (0.773–1.000)**
**2**	**Cer(d42:0)**	**127,504.392**	**0.90 (0.769–1.000)**
**3**	**TG(15:0_14:0_14:0)**	**3,788,045.717**	**0.90 (0.766–1.000)**
4	Cer(d18:0_24:0)	521,665.875	0.85 (0.673–1.000)
5	PC(18:2_18:2)	81,938,569.45	0.84 (0.654–1.000)
6	ChE(24:1)	52,345.314	0.83 (0.650–1.000)
7	PC(41:2)	33,392.237	0.83 (0.645–1.000)
8	BiotinylPE(30:3)	6,185,556.894	0.83 (0.602–1.000)
9	LPC(12:0)	379,006.021	0.79 (0.577–1.000)
10	Hex1Cer(t42:1 + O)	854,682.452	0.79 (0.562–1.000)
11	MePC(40:8e)	7,939,029.972	0.78 (0.531–1.000)
12	ChE(20:1)	66,948.94	0.77 (0.549–0.991)
13	MePC(34:6e)	1,029,943.584	0.77 (0.536–1.000)

*Lipids with top three AUC were selected as final candidate predictors (boldfaced notations)

Abbreviations: AUC, area under the ROC curve; CI; confidential interval; ROC, receiver operating characteristic

MS/MS for [SM(d35:1) + H]+, [Cer(d42:0) + HCOO]-, and [TG (15:0_14:0_14:0) + NH4] + demonstrated product ion peaks corresponding to phosphocholine, several fragments compatible with fragmentation of Cer(d42:0) with concomitant oxidation reaction, two fragments produced by neutral loss of fatty acid (FA)(14:0) or FA (15:0) from TG (15:0_14:0_14:0), respectively (Additional file 1, Supplemental Fig. 3). Consequently, the annotations of the final candidates by LipidSearch™ software were consistent with the results of MS/MS.

Among these three candidate predictors, SM(d35:1) was found to be positively correlated with Cer(d42:0) (Spearman’s rank correlation coefficient [rS] = 0.621, *P* = 0.004), TG (15:0_14:0_14:0) was inversely correlated with SM(d35:1) (rS = − 0.553, *P* = 0.013), and TG (15:0_14:0_14:0) was weakly inversely correlated with Cer(d42:0) (rS = − 0.353, *P* = 0.127) (Additional file 1, Supplemental Fig. 4).

### Validation of recurrence prediction ability among the final lipid candidates

Table 3 shows the sensitivity, specificity, and accuracy of the final candidate lipid predictors compared with the conventional pathological prognostic factors, lymph node metastasis, and blood vessel invasion, which were identified as significant recurrent factors in this cohort. Sensitivity of all three candidate lipid predictors is superior to that of lymph node metastasis. Patients with lymph node metastasis (all of them were hilar or lobar lymph node metastasis) corresponded to those in stage II. Among the recurrent group in this study cohort, half of the study population had stage I, whereas the other half had stage II. As lymph node metastasis can be detected among stage II cases, the sensitivity of lymph node metastasis was consequently lower than those of three candidate lipid predictors, which detected both stage I and stage II. Hence, these three predictors were superior to lymph node metastasis for patient screening. When comparing the candidate lipid predictors and blood vessel invasion, only SM(d35:1) showed prediction abilities higher or equal to those of blood vessel invasion in all validation points. Therefore, we propose SM(d35:1) as the most hopeful candidate for recurrence prediction.

**Table 3:** Comparison of sensitivity, specificity, and accuracy among the three final candidate predictors and conventional histopathological prognostic factors

Predictors for recurrence	Sensitivity	Specificity	Accuracy
Candidate lipid predictors			
SM(d35:1)*	1.00	0.80	0.90
Cer(d42:0)	0.90	0.70	0.80
TG(15:0_14:0_14:0)	1.00	0.70	0.85
Pathological prognostic factors			
Lymph node metastasis	0.50	1.00	0.75
Blood vessel invasion	0.90	0.80	0.85

*SM(d35:1) showed the most excellent prediction ability

Abbreviations: Cer, ceramide; SM, sphingomyelin; TG, triglyceride

## Discussion

In this study, candidate lipid predictors for lung adenocarcinoma recurrence after a radical surgery were retrospectively screened, and SM(d35:1) was found as the most prominent predictor, showing that the prediction ability was superior to that of conventional pathological prognostic factors in this small cohort.

The average total lipid level was significantly high in the recurrent group in this study. Furthermore, the number of increased lipid species was considerably higher than that of decreased lipid species in the recurrent group. These results were consistent with that of previous studies that showed an accelerated lipid synthesis in cancer cells, contributing to tumor phenotypes, such as cellular membrane building, stimulation of signaling pathways for growth and proliferation or survival under hypoxic conditions by supporting glycolysis [17, 18]. Increased total lipid level in the recurrent group may be biologically plausible because the aggressiveness may be supported by accelerated lipid synthesis.

The number of SM(d35:1) and Cer(d42:0), two of final candidate predictors, were increased in the recurrent group. SM and Cer are major bioactive components of lipid rafts on the cellular membrane [23]. SM is synthesized from Cer by SM synthase (SMS), which transfers the phosphocholine head group from phosphatidylcholine to Cer and results in concomitantly producing diacylglycerol (DAG). SM reconversion to Cer is catalyzed by sphingomyelinase (SMase) [23]. Increased SM abundance and SMS activity have been reported to play a critical role in cell proliferation and survival in several cancer types [23–26]. With regard to lung cancer, metabolic changes in sphingolipids are suggested to correlate with chemoresistance phenotype [27], and the total SM level in cancer tissues is reportedly lower than that of the normal lung tissue in patients with NSCLC [28]. This is speculated in the report that decreased SM abundance in lung cancer tissues may be attributable to high consumption of serine precursor by highly proliferating cancer cells [28]. Cer accumulation in the lungs has been suggested to participate in both cell apoptosis and tumorigenesis under cigarette smoke-induced oxidative stress [29]. Taking together these knowledge and significant positive correlation between SM(d35:1) + H and Cer(d42:0) in this study, increased synthesis flow of Cer toward SM in the recurrent group was suggested. Actually, significant increase on the total SM (*P* = 0.044) level and increased tendency on total Cer (*P* = 0.098) and DAG (*P* = 0.157) levels in the recurrent group were observed in this study cohort (Additional file 1, Supplemental Fig. 5). This result supports the suggestion of strong synthesis flow of Cer toward SM. The SM and Cer levels were not compared between the tumor tissues and normal lung tissues in this study, because normal lung tissue samples were lacking. Nonetheless, increased SM(d35:1) and Cer(d42:0) in the recurrent group in this study is consistent with previous studies [23–26, 28, 29] based on the following explanation: among lung adenocarcinomas with high SM and Cer consumption, cases that can maintain increased SM and Cer synthesis have highly aggressive phenotypes, resulting in recurrence.

Decreased TG (15:0_14:0_14:0) in the recurrent group was also included in the final candidate predictors. Although TG abundance in the lung cancer tissue has not yet been explored to date, TG level in colon cancer is reported to be lower as the disease progresses, suggesting that energy supply for colon cancer with higher degree of malignancy may depend on TG hydrolysis [30]. Inconsistent with the previous study [30], the total TG level in this study revealed no significant difference between the non-recurrent and recurrent groups (*P* = 0.350). Possible explanation for decreased TG (15:0_14:0_14:0) in the recurrent group is that aggressive recurrent lung adenocarcinoma that may preferably consume specific TG species for energy supply.

The difficulty of predicting lung cancer recurrence using histopathological prognostic factors may be partly attributed to subjective judgement. In addition, although the degree of histopathological prognostic factors widely varies, their judgements have been performed qualitatively [8, 10–16]; thereby, these methods may hinder accurate recurrence prediction and its retrospective validation. Conversely, excellent prediction ability of SM(d35:1) that is superior to histopathological factors was considered for its high objectivity and quantitative values. Actually, it was difficult to predict recurrent prognosis objectively from the conventional histopathological images of papillary-type adenocarcinoma, the most popular tissue subtype, with no significant difference between representative recurrent and non-recurrent cases, whereas the mass spectrum intensities of [SM(d35:1) + H]^+^ were markedly higher in the recurrent case to help recurrence prediction (Additional file 1, Supplemental Fig. 6). Furthermore, as high SM(d35:1) level was detected in all recurrent cases, including stage I and stage II cases, with high specificity and accuracy, SM(d35:1) was considered to be widely applicable for recurrence prediction in postoperative patients who underwent radical surgery.

Several limitations in this study should be acknowledged. First, this retrospective study is performed on a small sample size due to difficulty of obtaining frozen surgical specimens with clinical information that meet our inclusion criteria; thereby, verifying the reproducibility of using other validation cohorts was difficult. Thus, identified lipid predictors did not exceed above the “candidate” levels, and further large cohort studies should be conducted to validate candidate predictors identified in this study as rigid predictors for lung adenocarcinoma recurrence.

Because a large number of candidate lipid species (2595 species) relative to the small number of sample size (20 cases) were screened for candidate predictors, one candidate that shows near-perfect discrimination ability can be bound to be identified. Third, adjacent normal lung tissue samples were lacking, hence the difference between the abundance of the identified candidate lipid predictors in the normal lung tissue of the recurrent group and that of the non-recurrent group was not able to be compared. Fourth, because the non-recurrent group in this study included five cases that received adjuvant chemotherapy, the non-recurrent group may possibly include the recurrence high-risk cases; among them, recurrence might be prevented by adjuvant chemotherapy. Moreover, the non-recurrent group in this study included two cases with recurrence prediction positive for SM(d35:1) (Additional file 1, Supplemental Fig. 7). Among the two cases, one patient received adjuvant chemotherapy and the other did not. The former case may be considered as highly at risk for recurrence, which was prevented by adjuvant chemotherapy. The latter may be an exceptional case that cannot be ruled out by SM(d35:1). Fifth, because LC–MS/MS is not a universal examination in the clinical field, examining a large number of surgical specimens for recurrence prediction using LC–MS/MS is difficult. To utilize the findings of this study in a clinical field, lipid predictors should be replaced with other molecules that can be examined by universal methods, such as immunohistochemistry of SMS or SMase involved in the SM metabolism. Additionally, the sample cohort in this study included histopathological type of adenocarcinoma only. As a topic for future study, squamous cell carcinoma, a major histological subtype behind adenocarcinoma, should be explored for recurrent predictors through the lipidomic approach.

## Conclusions

We propose that SM(d35:1) is a hopeful candidate predictor for lung adenocarcinoma recurrence after a radical surgery. Our findings provide novel insights on the mechanisms of lung adenocarcinoma recurrence and can contribute to the development of precise recurrence prediction and qualified postoperative therapeutic strategy for lung adenocarcinoma.

## Supplementary information

**Additional file 1: Supplemental Table.** Weights of the frozen tissue samples. Each weight of the frozen tissue samples was measured using Sartorius analytical lab balance CPA224S (Sartorius AG, Göttingen, Germany) prior to lipid extraction. **Supplemental Fig. 1.** Recurrence-free survival (RFS) curve of the recurrent group. The 1-year and 2-year RFS rate of the recurrent group was 50 and 20% with the median RFS time of 12.5 (range, 9–38) months. **Supplemental Fig. 2.** Principal component analysis of 2595 identified lipid species. The recurrent group showed partial separations on the first three principal components. **Supplemental Fig. 3.** Tandem mass spectrometry (MS/MS) of the final candidate lipid species. Product ion spectra of MS/MS for (A) [SM(d35:1) + H]^+^, (B) [Cer(d42:0) + HCOO]^−^, and (C) [TG(15:0_14:0_14:0) + NH4]^+^ are shown. The product ion spectra showed peaks corresponding to (A) phosphocholine, (B) several fragments that are compatible with Cer(d42:0) fragmentation with concomitant oxidation reaction, (C) two fragments that are produced by neutral loss of FA(14:0) or FA(15:0) from TG(15:0_14:0_14:0). **Supplemental Fig. 4.** Spearman’s rank correlation analysis among the final three candidate predictors. Positive correlation between SM(d35:1) and Cer(d42:0), inverse correlation between TG(15:0_14:0_14:0) and SM(d35:1), weak inverse correlation between TG(15:0_14:0_14:0) and Cer(d42:0) were seen. Spearman’s rank correlation coefficients and *P*-values for significance are presented. **Supplemental Fig. 5.** Comparisons of the total levels of SM, Cer and DAG between the non-recurrent and recurrent groups. Significant increase on the total SM (*P* = 0.044) level and increasing tendency of the total Cer (*P* = 0.098) and DAG (*P* = 0.157) levels in the recurrent group were observed. **Supplemental Fig. 6.** Histopathological image, mass spectrum of [SM(d35:1) + H]^**+**^ and [PC(12:0_12:0) + H]^+^ from representative recurrent and non-recurrent cases. Hematoxylin-eosin staining of recurrent and non-recurrent cases (upper panel) showed typical papillary-type adenocarcinoma with no significant difference between the two cases, whereas mass spectrum intensities of [SM(d35:1) + H]^+^ (middle panel) were markedly higher in the recurrent case than that of the non-recurrent case to help recurrence prediction. A monoisotopic peak and two isotopic peaks of [SM(d35:1) + H]^+^ that prove high mass resolution in this study were detected in the respective cases. Monoisotopic peaks of [PC(12:0_12:0) + H]^+^ used for normalizing the monoisotopic peaks of [SM(d35:1) + H]^+^ intensity in respective case are shown (bottom panel). The lipid profiles of the two cases can be identified as recurrent case 3 and non-recurrent case 3 respectively in Additional file 2. **Supplemental Fig. 7.** The relationship between recurrence prediction using SM(d35:1) and medical history of the adjuvant chemotherapy. The non-recurrent group included one case with positive recurrence prediction using SM(d35:1) and medical history of adjuvant chemotherapy (described in red). This case may correspond to the cases highly at risk of recurrence that was prevented by the adjuvant chemotherapy.

**Additional file 2.** The full list of identified 2595 lipid species.

## Data Availability

The dataset supporting the conclusions of this article is included within the Additional files.

## Abbreviations

AUC: Area under the ROC curve
BiotinylPE: Biotinyl-phosphoethanolamine
Cer: Ceramide
ChE: Cholesterol ester
DAG: Diacylglycerol
FA: Fatty acid
Hex1Cer: Monohexosylceramide
LC–MS/MS: Liquid chromatography–tandem mass spectrometry
LPC: Lysophosphatidylcholine
MePC: Monoether phosphatidylcholine
NSCLC: Non-small cell lung cancer
PC: Phosphatidylcholine
PCA: Principal component analysis
RFS: Recurrent-free survival
ROC: Receiver operating characteristic
rS: Spearman’s rank correlation coefficient
SM: Sphingomyelin
SMase: Sphingomyelinase
SMS: Sphingomyelin synthase
STAS: Spread through air space
TG: Triglyceride
TNBC: Triple-negative breast cancer
