# Supplementary material for: Sphingomyelin(d35:1) as a novel predictor for lung adenocarcinoma recurrence after a radical surgery: a case-control study
Source: BMC Cancer. 2020 Aug 24;20:800. doi: 10.1186/s12885-020-07306-1 (PMC7446133; doi:10.1186/s12885-020-07306-1)
Supplement: Supplementary file 1 — Additional file 1: Supplemental Table. Weights of the frozen tissue samples. Each weight of the frozen tissue samples was measured using Sartorius analytical lab balance CPA224S (Sartorius AG, Göttingen, Germany) prior to lipid extraction. Supplemental Fig. 1. Recurrence-free survival (RFS) curve of the recurrent group. The 1-year and 2-year RFS rate of the recurrent group was 50 and 20% with the median RFS time of 12.5 (range, 9–38) months. Supplemental Fig. 2. Principal component analysis of 2595 identified lipid species. The recurrent group showed partial separations on the first three principal components. Supplemental Fig. 3. Tandem mass spectrometry (MS/MS) of the final candidate lipid species. Product ion spectra of MS/MS for (A) [SM(d35:1) + H]+, (B) [Cer(d42:0) + HCOO]−, and (C) [TG(15:0_14:0_14:0) + NH4]+ are shown. The product ion spectra showed peaks corresponding to (A) phosphocholine, (B) several fragments that are compatible with Cer(d42:0) fragmentation with concomitant oxidation reaction, (C) two fragments that are produced by neutral loss of FA(14:0) or FA(15:0) from TG(15:0_14:0_14:0). Supplemental Fig. 4. Spearman’s rank correlation analysis among the final three candidate predictors. Positive correlation between SM(d35:1) and Cer(d42:0), inverse correlation between TG(15:0_14:0_14:0) and SM(d35:1), weak inverse correlation between TG(15:0_14:0_14:0) and Cer(d42:0) were seen. Spearman’s rank correlation coefficients and P-values for significance are presented. Supplemental Fig. 5. Comparisons of the total levels of SM, Cer and DAG between the non-recurrent and recurrent groups. Significant increase on the total SM (P = 0.044) level and increasing tendency of the total Cer (P = 0.098) and DAG (P = 0.157) levels in the recurrent group were observed. Supplemental Fig. 6. Histopathological image, mass spectrum of [SM(d35:1) + H]+ and [PC(12:0_12:0) + H]+ from representative recurrent and non-recurrent cases. Hematoxylin-eosin staining of recurrent an [file 12885_2020_7306_MOESM1_ESM.pptx]

## Slide 1
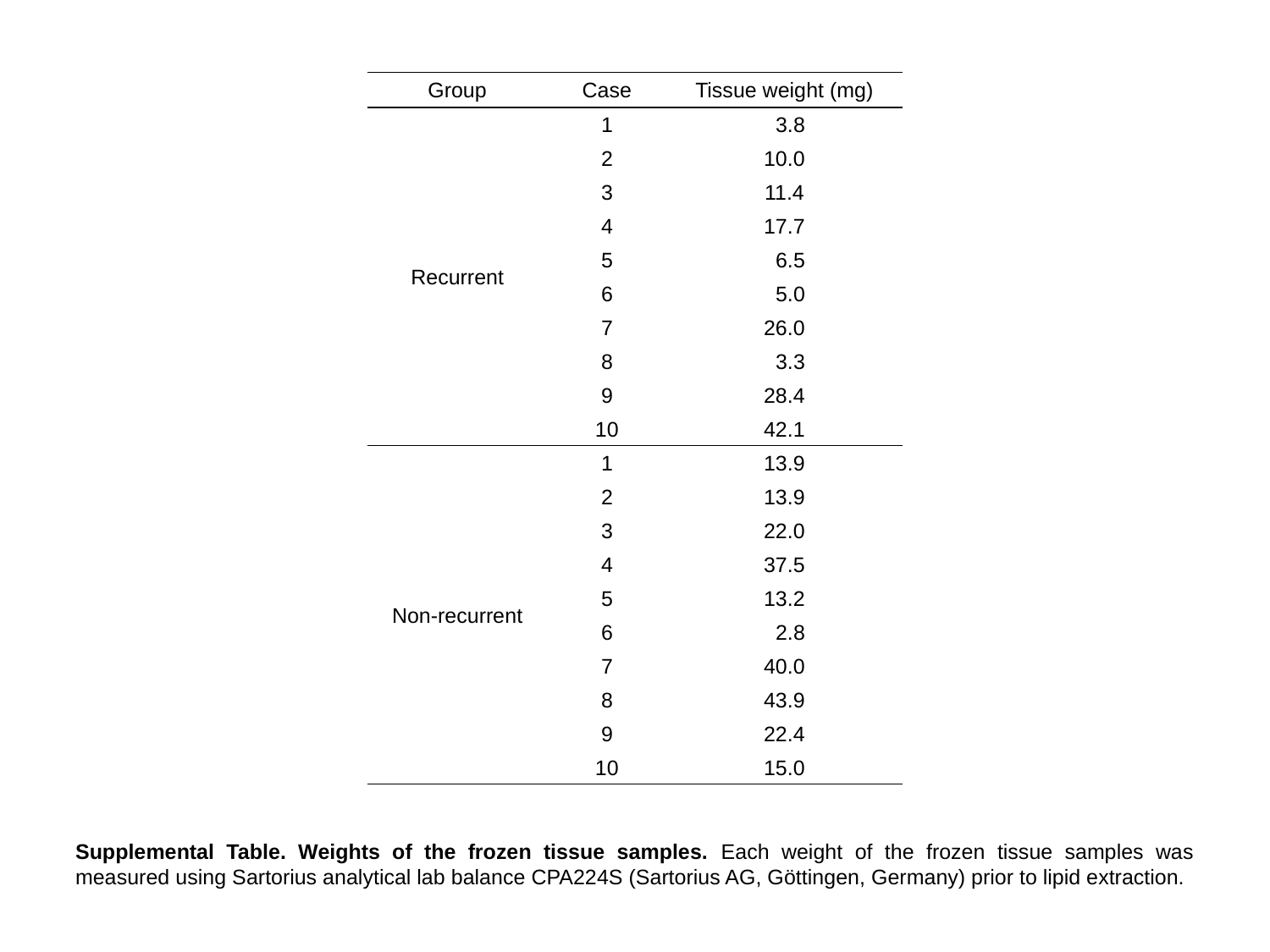

| Group | Case | Tissue weight (mg) |
| --- | --- | --- |
| Recurrent | 1 | 3.8 |
| | 2 | 10.0 |
| | 3 | 11.4 |
| | 4 | 17.7 |
| | 5 | 6.5 |
| | 6 | 5.0 |
| | 7 | 26.0 |
| | 8 | 3.3 |
| | 9 | 28.4 |
| | 10 | 42.1 |
| Non-recurrent | 1 | 13.9 |
| | 2 | 13.9 |
| | 3 | 22.0 |
| | 4 | 37.5 |
| | 5 | 13.2 |
| | 6 | 2.8 |
| | 7 | 40.0 |
| | 8 | 43.9 |
| | 9 | 22.4 |
| | 10 | 15.0 |
Supplemental Table. Weights of the frozen tissue samples. Each weight of the frozen tissue samples was measured using Sartorius analytical lab balance CPA224S (Sartorius AG, Göttingen, Germany) prior to lipid extraction.

## Slide 2
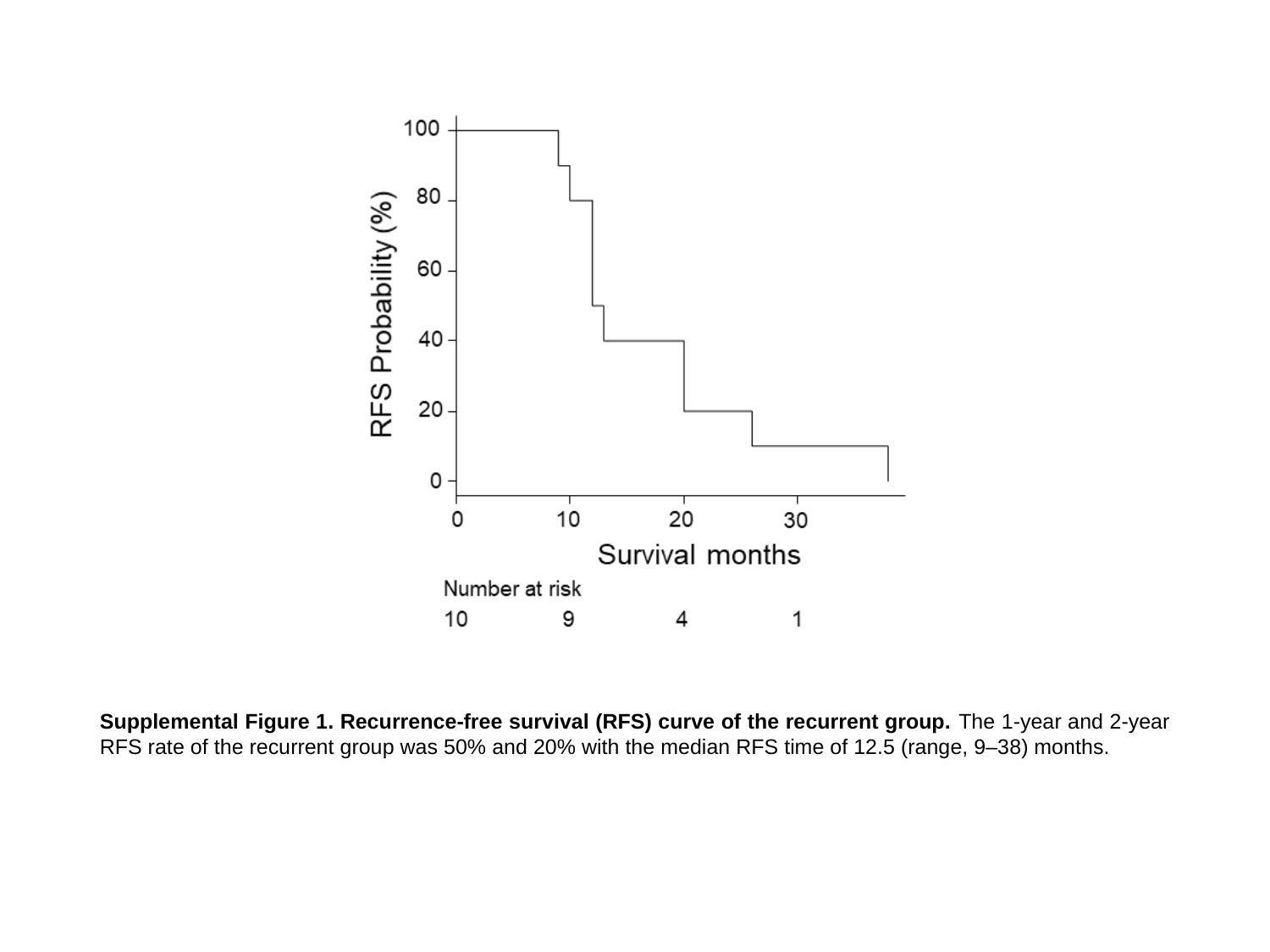

Supplemental Figure 1. Recurrence-free survival (RFS) curve of the recurrent group. The 1-year and 2-year RFS rate of the recurrent group was 50% and 20% with the median RFS time of 12.5 (range, 9–38) months.

## Slide 3
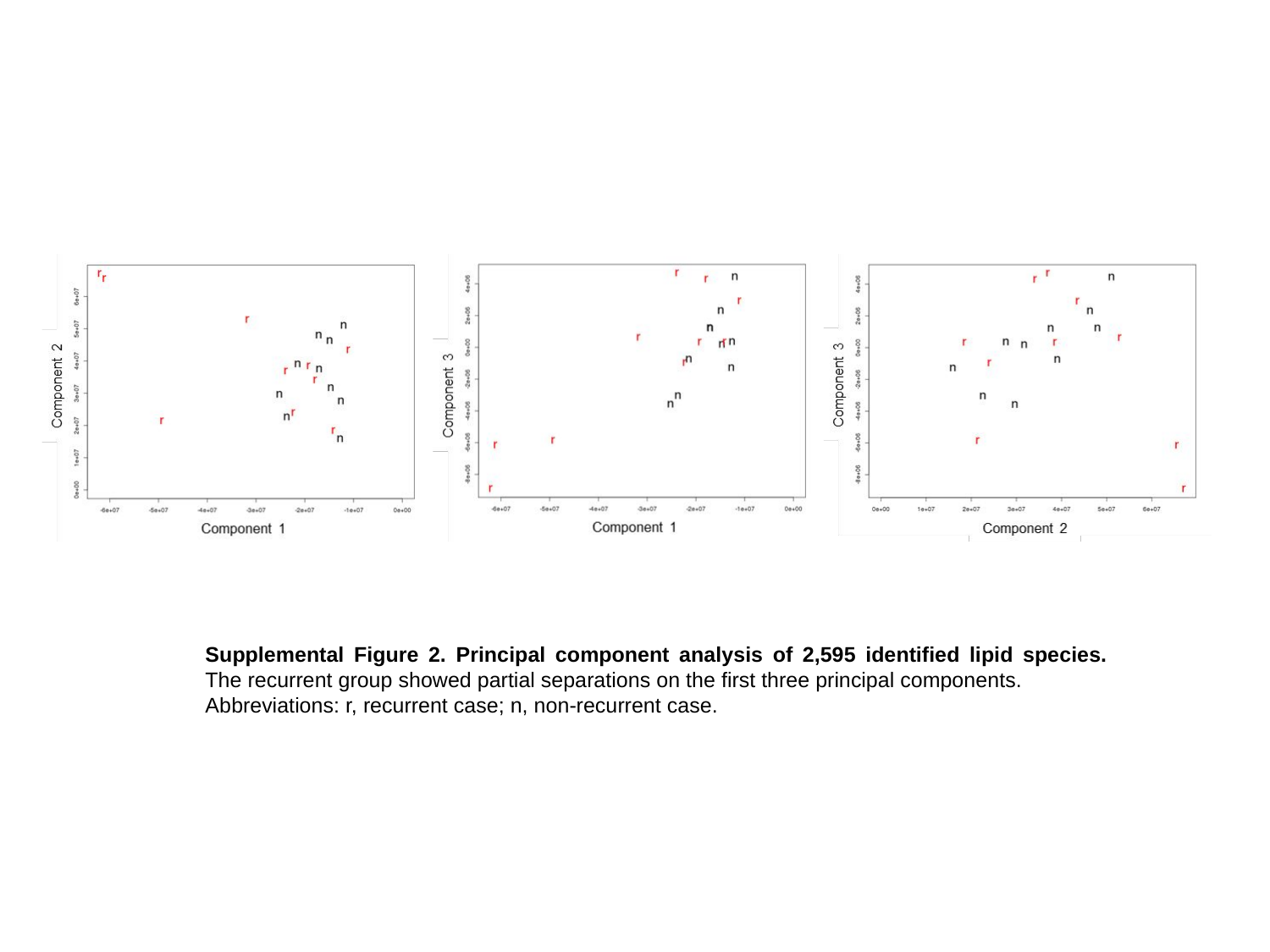

Supplemental Figure 2. Principal component analysis of 2,595 identified lipid species. The recurrent group showed partial separations on the first three principal components.
Abbreviations: r, recurrent case; n, non-recurrent case.

## Slide 4
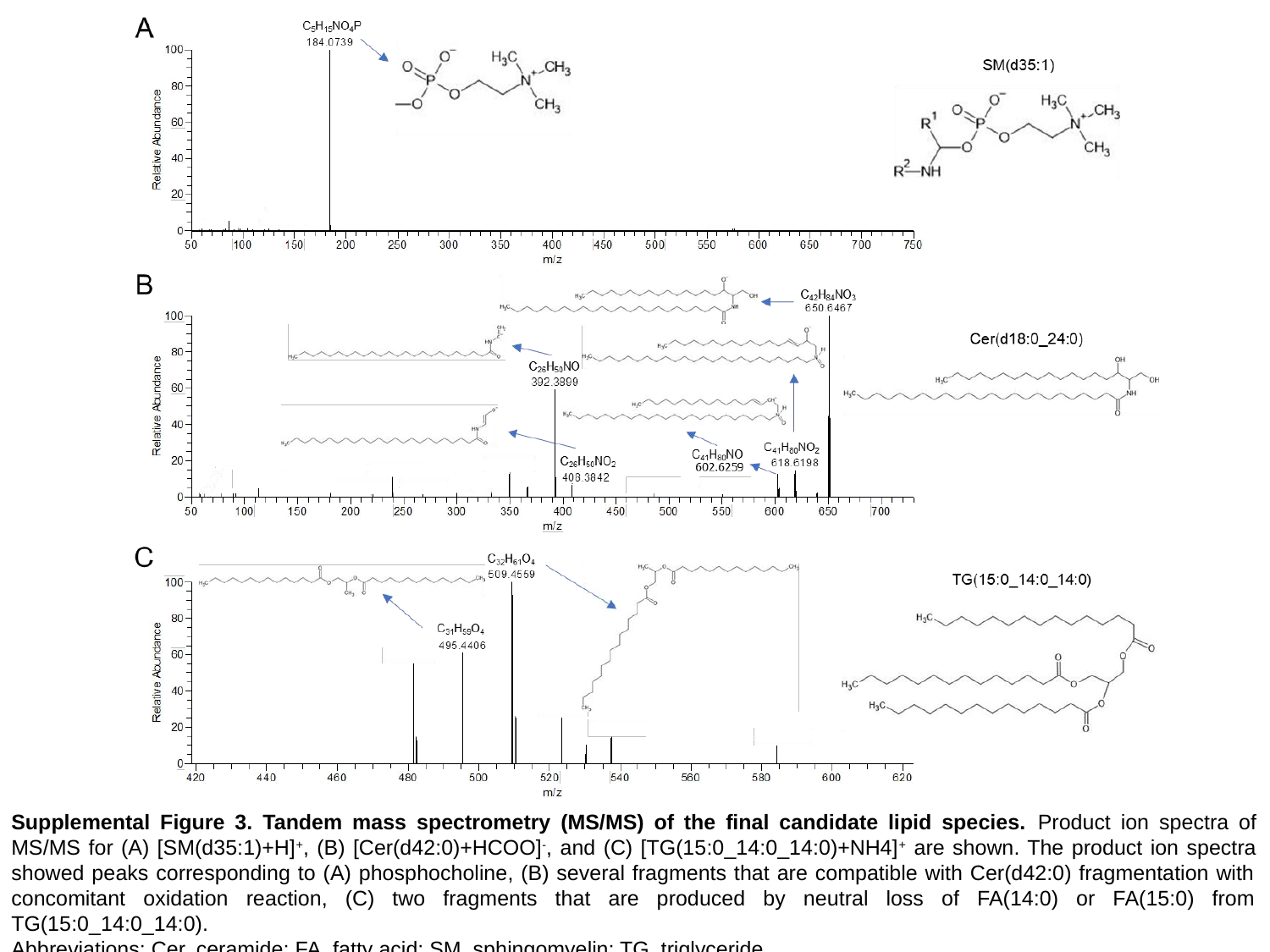

Supplemental Figure 3. Tandem mass spectrometry (MS/MS) of the final candidate lipid species. Product ion spectra of MS/MS for (A) [SM(d35:1)+H]+, (B) [Cer(d42:0)+HCOO]-, and (C) [TG(15:0_14:0_14:0)+NH4]+ are shown. The product ion spectra showed peaks corresponding to (A) phosphocholine, (B) several fragments that are compatible with Cer(d42:0) fragmentation with concomitant oxidation reaction, (C) two fragments that are produced by neutral loss of FA(14:0) or FA(15:0) from TG(15:0_14:0_14:0).
Abbreviations: Cer, ceramide; FA, fatty acid; SM, sphingomyelin; TG, triglyceride.

## Slide 5
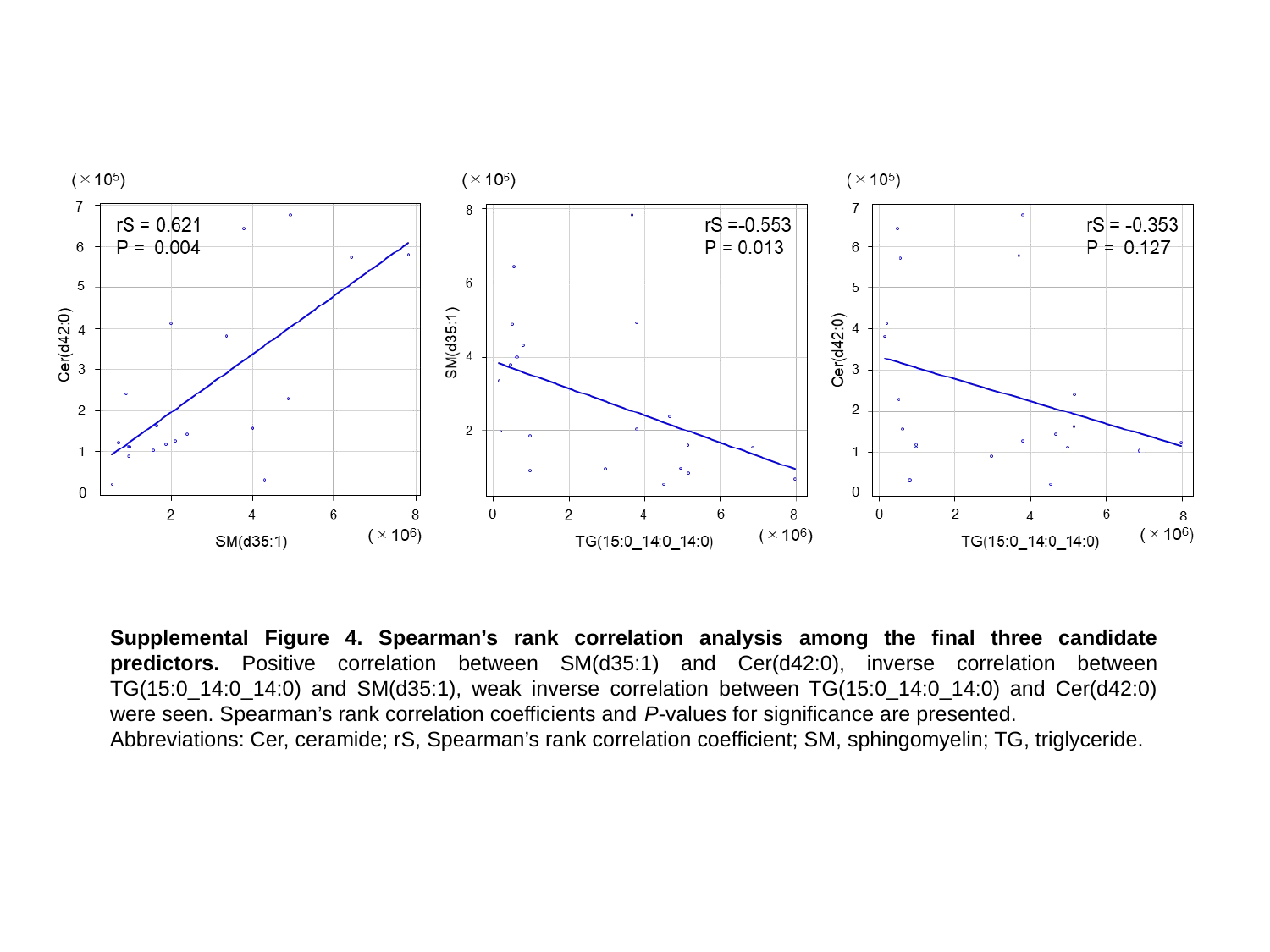

Supplemental Figure 4. Spearman’s rank correlation analysis among the final three candidate predictors. Positive correlation between SM(d35:1) and Cer(d42:0), inverse correlation between TG(15:0_14:0_14:0) and SM(d35:1), weak inverse correlation between TG(15:0_14:0_14:0) and Cer(d42:0) were seen. Spearman’s rank correlation coefficients and P-values for significance are presented.
Abbreviations: Cer, ceramide; rS, Spearman’s rank correlation coefficient; SM, sphingomyelin; TG, triglyceride.

## Slide 6
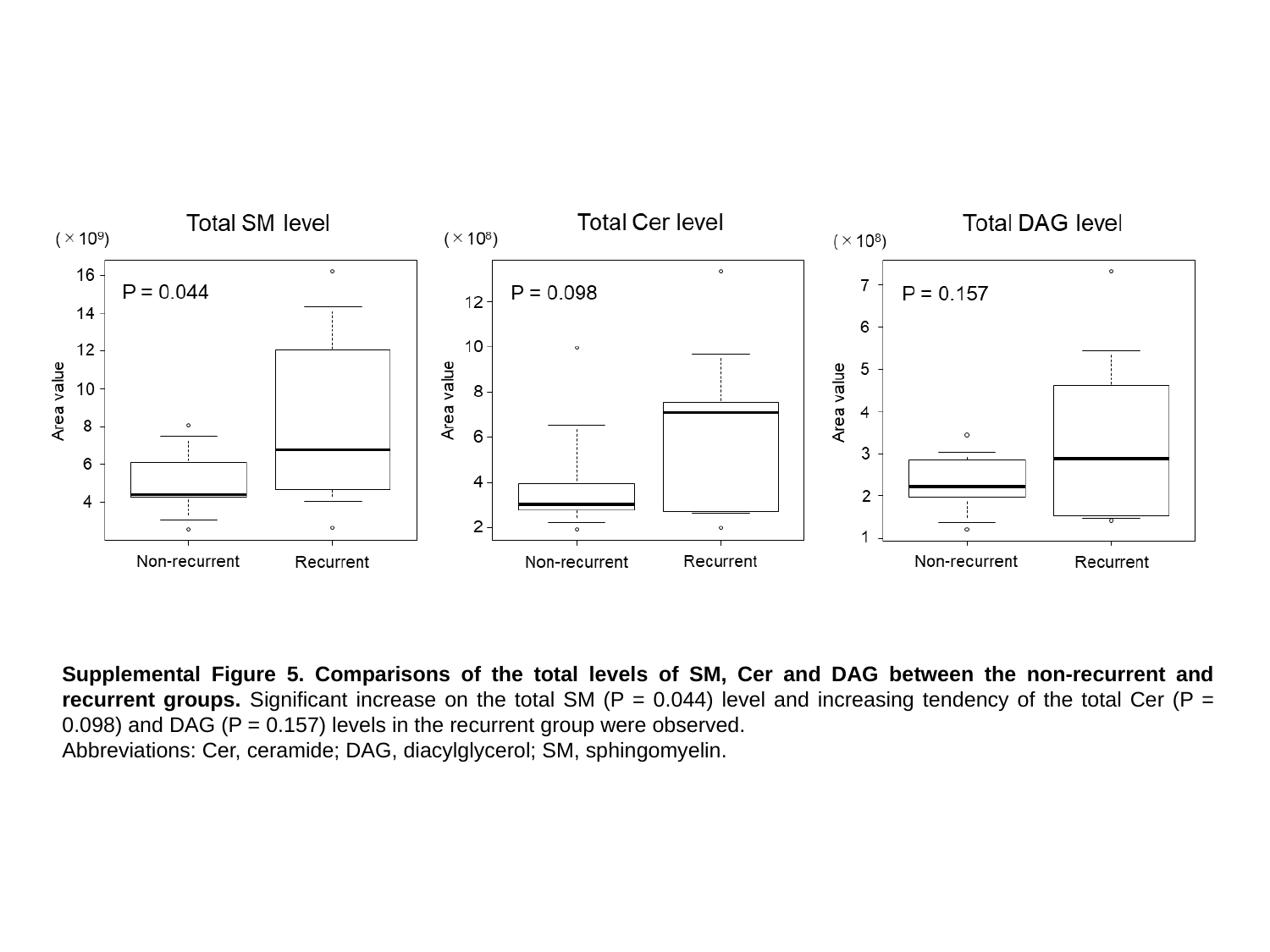

Supplemental Figure 5. Comparisons of the total levels of SM, Cer and DAG between the non-recurrent and recurrent groups. Significant increase on the total SM (P = 0.044) level and increasing tendency of the total Cer (P = 0.098) and DAG (P = 0.157) levels in the recurrent group were observed.
Abbreviations: Cer, ceramide; DAG, diacylglycerol; SM, sphingomyelin.

## Slide 7
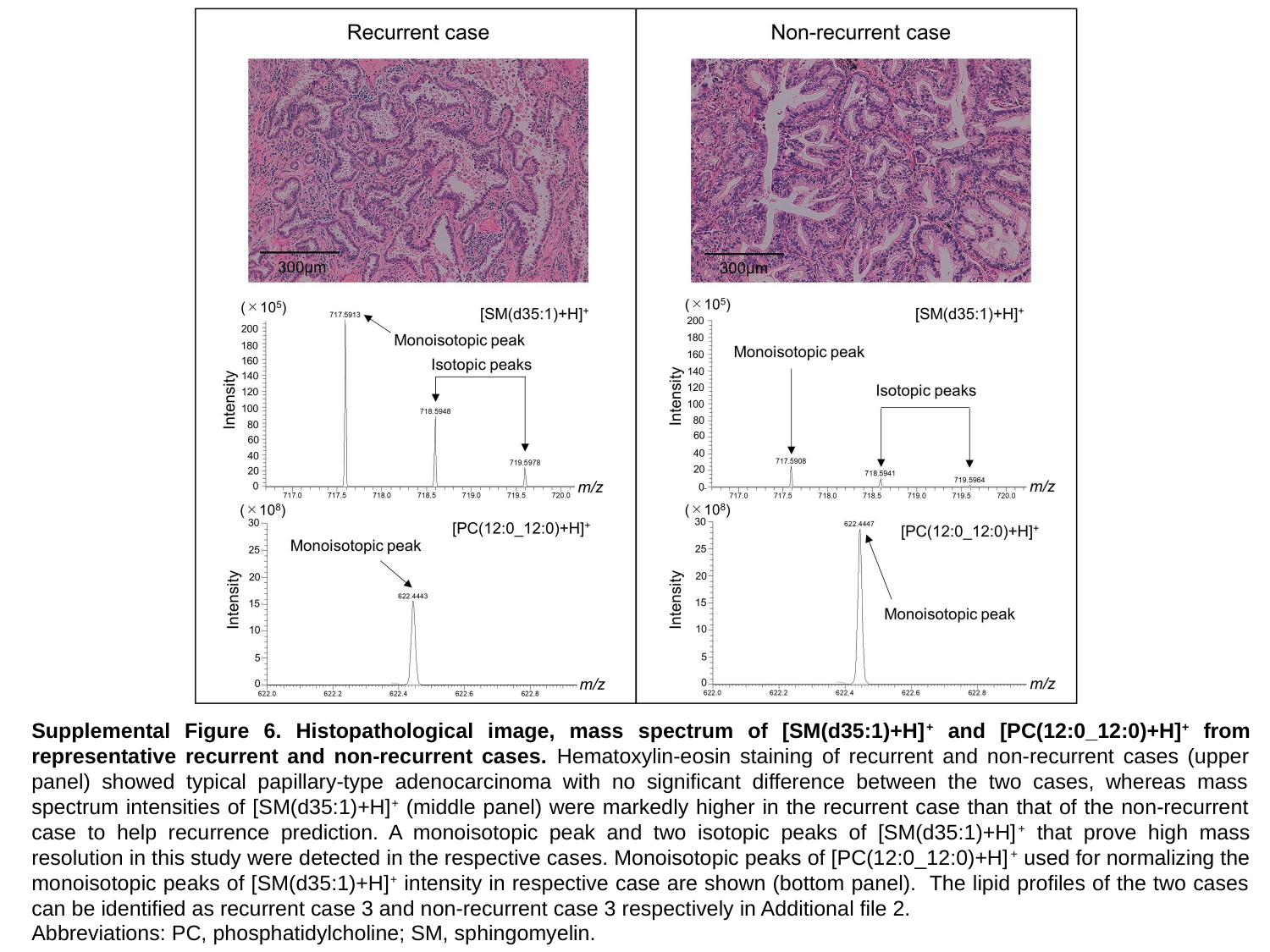

Supplemental Figure 6. Histopathological image, mass spectrum of [SM(d35:1)+H]+ and [PC(12:0_12:0)+H]+ from representative recurrent and non-recurrent cases. Hematoxylin-eosin staining of recurrent and non-recurrent cases (upper panel) showed typical papillary-type adenocarcinoma with no significant difference between the two cases, whereas mass spectrum intensities of [SM(d35:1)+H]+ (middle panel) were markedly higher in the recurrent case than that of the non-recurrent case to help recurrence prediction. A monoisotopic peak and two isotopic peaks of [SM(d35:1)+H]+ that prove high mass resolution in this study were detected in the respective cases. Monoisotopic peaks of [PC(12:0_12:0)+H]+ used for normalizing the monoisotopic peaks of [SM(d35:1)+H]+ intensity in respective case are shown (bottom panel). The lipid profiles of the two cases can be identified as recurrent case 3 and non-recurrent case 3 respectively in Additional file 2.
Abbreviations: PC, phosphatidylcholine; SM, sphingomyelin.

## Slide 8
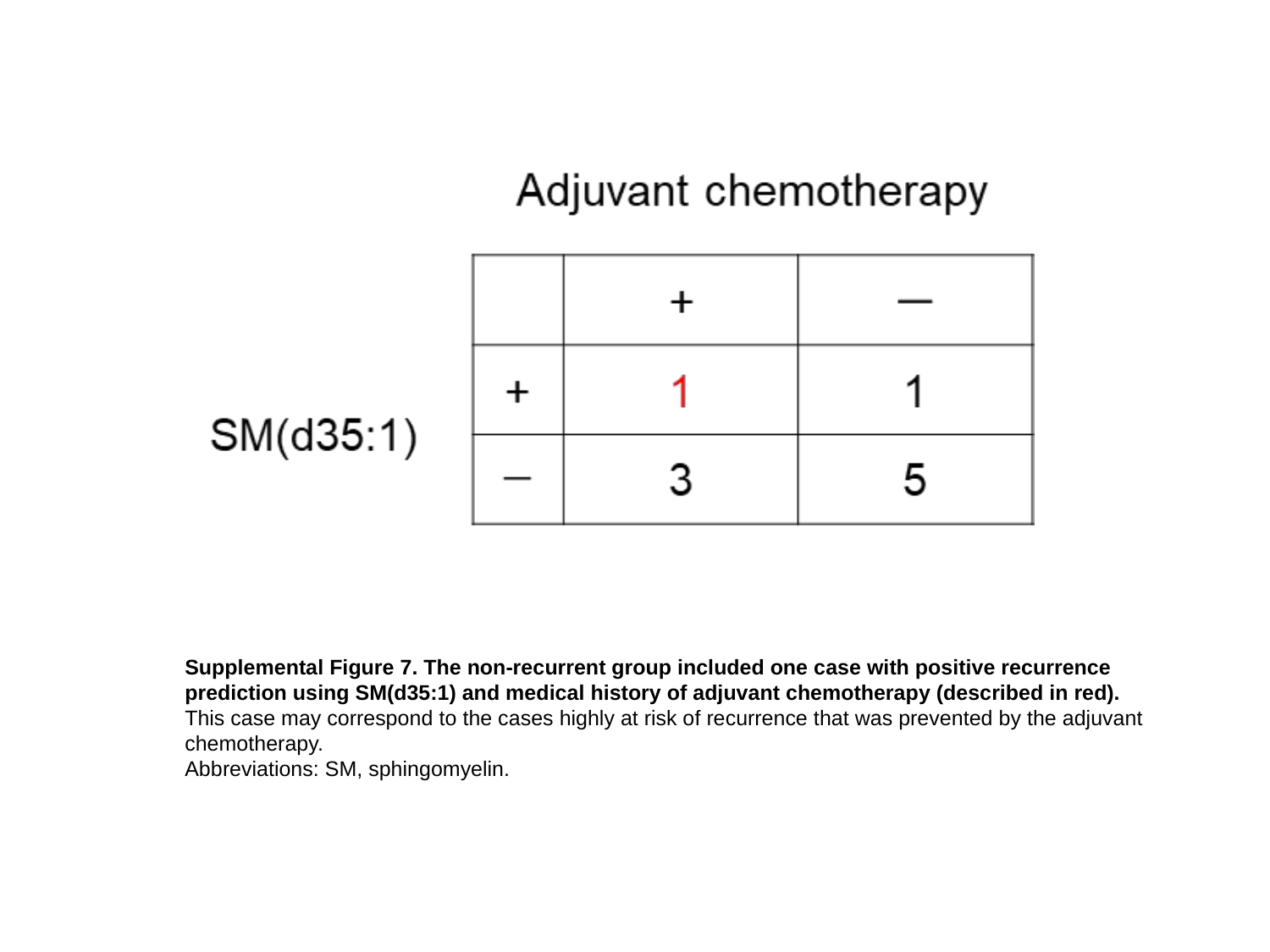

Supplemental Figure 7. The non-recurrent group included one case with positive recurrence prediction using SM(d35:1) and medical history of adjuvant chemotherapy (described in red). This case may correspond to the cases highly at risk of recurrence that was prevented by the adjuvant chemotherapy.
Abbreviations: SM, sphingomyelin.
